# NAV-001, a high-efficacy antibody-drug conjugate targeting mesothelin with improved delivery of a potent payload by counteracting MUC16/CA125 inhibitory effects

**DOI:** 10.1371/journal.pone.0285161

**Published:** 2023-05-17

**Authors:** Nicholas C. Nicolaides, J. Bradford Kline, Luigi Grasso

**Affiliations:** Navrogen Inc., Cheyney, PA, United States of America; BRAC University, BANGLADESH

## Abstract

Subsets of tumor-produced cell surface and secreted proteins can bind to IgG_1_ type antibodies and suppress their immune-effector activities. As they affect antibody and complement-mediated immunity, we call these proteins humoral immuno-oncology (HIO) factors. Antibody-drug conjugates (ADCs) use antibody targeting to bind cell surface antigens, internalize into the cell, then kill target cells upon liberation of the cytotoxic payload. Binding of the ADC antibody component by a HIO factor may potentially hamper ADC efficacy due to reduced internalization. To determine the potential effects of HIO factor ADC suppression, we evaluated the efficacy of a HIO-refractory, mesothelin-directed ADC (NAV-001) and a HIO-bound, mesothelin-directed ADC (SS1). The HIO factor MUC16/CA125 binding to SS1 ADC was shown to have a negative effect on internalization and tumor cell killing. The MUC16/CA125 refractory NAV-001 ADC was shown to have robust killing of MUC16/CA125 expressing and non-expressing tumor cells *in vitro* and *in vivo* at single, sub-mg/kg dosing. Moreover, NAV-001-PNU, which contains the PNU-159682 topoisomerase II inhibitor, demonstrated good stability *in vitro* and *in vivo* as well as robust bystander activity of resident cells while maintaining a tolerable safety profile *in vivo*. Single-dose NAV-001-PNU demonstrated robust tumor regression of a number of patient-derived xenografts from different tumor types regardless of MUC16/CA125 expression. These findings suggest that identification of HIO-refractory antibodies to be used in ADC format may improve therapeutic efficacy as observed by NAV-001 and warrants NAV-001-PNU’s advancement to human clinical trials as a monotherapy to treat mesothelin-positive cancers.

## Introduction

Tumors employ a variety of mechanisms to avoid host immune responses and therapeutic-based killing. Several strategies have been developed to overcome such defense mechanisms including development of immune checkpoint inhibitors as well as other immune-mediated therapies including T-cells engineered with chimeric antigen targeting receptors (CAR-Ts), vaccines, bispecific antibodies and antibody-drug conjugates (ADCs). Recent findings have found that tumors can produce cell surface and secreted proteins, referred to as humoral immuno-oncology (HIO) factors, that can bind to antibodies and block their immune-effector activities. These effects occur via direct binding and blockade of antibody interactions with Fc-γ-activating receptors on natural killer (NK) and subsets of myeloid, dendritic and monocytic cells [1, 2]. These effects negatively impact antibody-dependent cellular cytotoxicity (ADCC) and complement-mediated cytotoxicity (CDC) against antibody-bound tumors [3–5]. Strategies to overcome humoral immunosuppression of antibody-based therapeutics include screening for those that are naturally not bound by HIO factors or by employing next generation antibody technologies not requiring immune-effector activity. The latter include the use of ADC formatted antibodies or those with modified amino acids critical for HIO factor binding, a process referred to as Block-Removed Immunoglobulin Technology (*BRITE*) [2].

The application of ADCs has improved the efficacy of several antibodies. However, the impact of HIO factor binding to ADCs has not been evaluated. Here we describe the suppressive effect of HIO factors on ADCs due to lower cellular uptake, a requisite for effective ADC-mediated killing [6]. Additionally, we describe the development of a novel ADC targeting mesothelin (MSLN), a cell surface protein that is over-expressed by a number of tumor types including mesothelioma, lung, pancreatic, ovarian, colorectal, cholangial, gastric and endometrial carcinomas [7, 8]. Importantly, several of these MSLN-expressing tumors have been found to produce HIO factors that can cause immunosuppression. In particular lung, ovarian, pancreatic, colorectal and mesothelioma cancers have been reported to express the immunosuppressive MUC16/CA125 HIO factor [9, 10]. NAV-001 is an anti-MLSN ADC consisting of a humanized IgG_1_ antibody chemically linked to the PNU-159682 topoisomerase II inhibitor. The antibody component consists of a humanized version of the YP218 monoclonal antibody discovered using standard hybridoma methods [11]. The antibody was selected for ADC development due to its inherent MUC16/CA125 non-binding properties. The NAV-001-PNU-159682 (referred herein as NAV-001-PNU) format was identified from empirical screening of various linker-cytotoxin formats against HIO-positive and -negative MSLN-expressing tumor cell lines. *In vitro*, the MUC16/CA125 refractory NAV-001-PNU was found to be more effective against MUC16/CA125 HIO-positive tumor cell lines in contrast to another MSLN-PNU ADC (SS1-PNU) that was bound by MUC16/CA125. This effect appeared to be due to reduced internalization. *In vivo*, NAV-001-PNU was also found to be highly effective against a number patient derived-xenografts (PDX) regardless of MUC16/CA125 expression. These novel concepts provide additional parameters to consider when developing ADCs, especially in cancers where the MUC16/CA125 HIO factor is overproduced. MUC16/CA125 is not the only HIO factor capable of binding IgG-type antibodies [12], therefore broad *in vitro* screening of ADC antibody components may be warranted against a panel of HIO-positive and -negative cancer lines to identify those that are most effective for antigen binding, internalization and target cell killing.

## Materials and methods

### Materials information

#### Antibodies and antibody-drug conjugates

Antibodies were acquired from vendors and through academic collaboration. Antibodies Ab-1, Ab-2 (humanized SS1), Ab-4 and Ab-5 were produced internally or purchased (Creative BioLabs). NAV-001 and Ab-1 anti-mesothelin antibodies were obtained from Drs. Mitchell Ho and Ira Pastan (National Cancer Institute) and was characterized as previously described [11, 13]. ADCs were made using the cytotoxic topoisomerase I inhibitor SN-38 and the topoisomerase II inhibitor PNU-159682. Conjugations were done under a controlled temperature environment, whereby equal amounts of antibody were treated with optimal amounts of tris(2-carboxyethyl)phosphine to partially reduce interchain cysteines, followed by addition of optimal molar PEG8-triazole-PABC-peptide-SN-38 (SN-38 ADC format) or MA-PEG4-VC-PAB-DMAE-PNU159682 (PNU ADC format) linkers followed by reoxidation. Purified conjugates were analyzed by Hydrophobic Interaction Chromatography (HIC-HPLC) and Size Exclusion Chromatography (SEC-HPLC) to determine drug-antibody-ratios (DAR) and antibody aggregation, respectively. The average DAR for SN-38 ADCs were 6 and PNU-159682 ADCs were 4. Both ADC formats had less than 3.0% aggregates.

#### Cell lines and patient-derived tumors

The human ovarian cancer cell line OVCAR-3, human gastric cancer NCI-N87, Chinese Hamster Ovary (CHO) and T-cell lymphoma Jurkat cells were purchased from ATCC and maintained in complete RPMI media (R7.5) containing 7.5% fetal bovine serum (Gibco), 1% L-glutamine and 1% penicillin-streptomycin. MUC16/CA125 shRNA OVCAR-3 knockdown lines were generated using similar constructs and methods as previously described [3]. Analysis of independent knockdown vectors and clones showed reproducible loss of MUC16/CA125 expression and similar levels of the MSLN cell surface antigen between the parental (OVCAR-3) and knockdown (OV-KD) lines. The representative OV-KD line was generated using the Mission Lentiviral TRCN0000262688 (Sigma-Aldrich) construct. Patient-derived tumor fragments (PDFs) used for PDX models were first chosen by mRNA expression profiling of MSLN and MUC16/CA125 from our vendor’s tumor bank (Charles River Labs). Highest expressing PDFs from various tumor types were then tested for protein expression by immunohistochemistry as described below. PDFs with the most homogeneous cell surface expression where then used for *in vivo* testing.

### Methods

#### Cytotoxicity assays

To test for direct and bystander ADC killing activity of target cell lines, assays were conducted in 96-well plates and carried out for 96 hours at 37°C in 5% CO_2_ in R7.5 media in triplicate. For direct ADC killing assays, target cells were trypsinized and seeded at 5,000 cells/well, grown for 48 hours, treated with varying amounts of ADC (0.010–250 ng/mL) then grown for an additional 96 hours. For bystander ADC killing, plates were seeded with 5,000 cells/well with the adherent MSLN-positive NCI-N87 cells, MSLN-negative CHO or no cells for 48 hours in R7.5 media. Wells were washed with Dulbecco’s phosphate buffered saline without Mg^++^ or Ca^++^ (DPBS) and replated with 50,000 Jurkat cells/mL in R7.5 with varying amounts of NAV-001-PNU (1–750 ng/mL) or free PNU-159682 (0.1–100 pM) and grown for 96 hours. For cell viability of direct ADC killing, wells were washed 3 times with DPBS then stained with crystal violet for 10 minutes, washed in distilled water 5 times, air dried, solubilized in 1% SDS and quantified on a Varioskan plate reader at 570 nm (ThermoScientific). For viability of non-adherent Jurkat bystander cells, culture supernatant containing Jurkat cells was collected and cell viability was measured using Cell Titer-Glo™ (Promega) bioluminescence following the manufacturer’s instructions and relative lumens (RLU) determined using a Varioskan plate reader. Percent cytotoxicity was calculated by the formula [1-(treated/untreated)] x 100%.

#### Enzyme-linked immunosorbent assays (ELISA)

ELISA assays were used to measure anti-MSLN antibody binding to MUC16/CA125, MSLN and human serum albumin (HSA, negative control) as previously described [3]. Briefly, 96-well plates were coated with 5,000 units/mL MUC16/CA125 (Lee Biosolutions), 100 ng/mL recombinant MSLN (Sino Biologicals) or 100 ng/mL HSA (Sigma) in 50 mM carbonate buffer, pH 9.5 overnight at 4°C. Plates were washed with 50 mM phosphate buffer, pH 7.2 (PB) and blocked in PB plus 5% bovine serum albumin (BSA) for 1 hour at room temperature. For anti-MSLN binding, wells were washed with PB, then probed with 2.5 μg/mL of biotinylated anti-MSLN antibody in PB plus 0.5% BSA for 1 hour at room temperature. For MUC16/CA125 competition of anti-MSLN antibody binding to MSLN protein, anti-MSLN antibody was added with 15,000 units/mL of MUC16/CA125 and probed as above. After primary probing, wells were washed with PB and secondary probed with 333 ng/mL streptavidin-horse radish peroxidase (HRP) in PB plus 0.5% BSA for 1 hour at room temperature, washed with PB, then incubated with 3,3′,5,5′-tetramethylbenzidine (TMB) substrate (ThermoFisher) for 10 minutes. Reactions were stopped with 0.1N H_2_SO_4_ and plates were analyzed for absorbance at 450 nm using a Varioskan plate reader. Antibodies were biotinylated using sulfo-tag conjugation (Meso Scale Diagnostics) as previously described with multiple independent lots generated to confirm reproducibility [3].

#### Internalization assays

Antibody internalization was measured using OVCAR-3 and OV-KD cells and the PHrodo™ Red Avidin diagnostic method following the manufacturer’s instructions (ThermoFisher). PHrodo Red-labelled antibodies were generated by adding 25 μg/mL of PHrodo Red Avidin to 25 μg/mL biotinylated or non-biotinylated (negative control) antibodies for 30 minutes at room temperature in microfuge tubes. Upon completion, reactions were centrifuged at 10,000 g for 2 minutes to remove aggregates. Target cells were seeded in triplicate at 10,000 cell/well in 96-well opaque plates and grown for 48 hours in R7.5 media at 37°C in 5% CO_2_. To monitor internalization, PHrodo Red-labelled antibodies and controls were added at a final concentration of 5 μg/mL to 4°C chilled R7.5 media. Next, PHrodo Red-labelled antibodies were added to appropriate wells and plates were incubated on ice for 1 hour to minimize immediate cellular uptake. Wells were then washed twice with 4°C DPBS to remove unbound PHrodo-Red or control antibody. Plates were incubated at 37°C in 5% CO_2_ and monitored for uptake via fluorescence over a 24-hour period using a Varioskan plate reader.

#### Immunohistochemistry (IHC)

To confirm MSLN and MUC16/CA125 expression in PDFs, we employed IHC using a rabbit anti-MSLN antibody that binds the same epitope as NAV-001 and a commercial rabbit anti-MUC16/CA125 antibody (Novus), respectively. Briefly, 5 μM sections of paraffin embedded tumor fragments were sectioned via microtome then adhered to glass slides. Sections were deparaffinized and prepared for antigen retrieval in boiling 10 mM sodium citrate, pH 6.0 for 10 minutes, then equilibrated with phosphate buffered saline-0.05% tween-20 (PBS-T). Sections were quenched for endogenous peroxidase activity using 0.3% peroxidase/methanol for 10 minutes and blocked for 1 hour in 10% goat serum in PBS-T. Next, slides were rinsed in PBS-T and probed for MSLN or MUC16/CA125 using 3 μg/mL of each primary antibody diluted in blocking buffer for 1.5 hours followed by PBS-T washing, secondary blocking for 1 hour and probing with 5 μg/mL of anti-rabbit-HRP conjugated secondary antibody for 1 hour. Control slides were incubated with no primary antibody. Slides were washed then developed using eBioscience™ DAB advanced chromogenic substrate as recommended by the manufacturer (ThermoFisher). Slides were hematoxylin counterstained, cover-slipped and analyzed for antigen expression under light microscopy using a Zeiss Axioplan widefield microscope. Membranous staining was considered positive.

#### NAV-001-PNU serum stability

To determine NAV-001-PNU serum stability, NAV-001-PNU was analyzed via bioassay and ELISA. For Bioassay, NAV-001-PNU or free PNU-159682 were diluted to 20 μg/mL in human, cynomolgus monkey, rat and mouse plasma and incubated 0–14 days at 37°C. Stressed plasma was then diluted to 100 ng/mL and added to MSLN-negative human A549 cells for 3 days and tested for viability via crystal violet staining at day 14 as described above. PNU-159682 concentration of 12 pg/mL represents 1% of free PNU in a 100 ng/mL concentration of NAV-001-PNU. For ELISA, same samples as above were tested for total IgG and ADC stability. Stock NAV-001-PNU was serially diluted in the various host sera ranging from 0 to 100 ng/mL. Stressed plasma samples were diluted to a theoretical concentration of 50 ng/mL. For total IgG ELISA, 96-well plates were coated with 2 μg/mL of a goat anti-human IgG (Southern Biotech) in 50 mM carbonate buffer, pH 9.5. For ADC ELISA, 96-well plates were coated with 2 μg/mL of a mouse anti-PNU antibody (Levena Biopharma) in carbonate buffer. Uncoated wells served as negative control for both assays. Plates were incubated overnight at 4°C then washed with PB and blocked with PB plus 5% BSA as described above. Next, wells were washed with PB and probed with stressed plasma samples or NAV-001-PNU standards for 1 hour at room temperature. Plates were washed and probed with 50 ng/mL of goat anti-human IgG-HRP (Bethyl Laboratories) in PB plus 0.5% BSA for 1 hour at room temperature, then washed and developed using TMB substrate for 15 minutes. Reactions were stopped with H_2_SO_4_ and analyzed for absorbance at 450 nm using a Varioskan plate reader. All samples were tested in triplicate.

#### Pharmacokinetic analysis

Pharmacokinetics (PK) of NAV-001-PNU was evaluated in 7–10 week-old female CD-1 mice by contract research with Biocytogen (Wakefield, MA). Animals were treated in replicas with the minimal effective doses of 0.25 and 0.75 mg/kg NAV-001-PNU via tail vein injection. Animals were observed under routine monitoring for any effects related to treatment that included mobility; food and water consumption; body weight gain/loss; grooming; peri-orbital bleeding and any other abnormalities. Blood samples were collected at pre-dose, 1, 24, 72, 168, and 336 hours after injection by cardiac puncture using EDTA-coated syringes and transferred to EDTA-coated tubes. For each time point, a single animal was terminally bled. Blood was collected into EDTA-coated tubes, centrifuged 2,000 g at 4°C, and plasma supernatants were collected and transferred into fresh tubes, quantified for total protein and stored at -80°C until ELISA analysis of NAV-001-PNU total antibody and ADC concentrations was conducted. Noncompartmental PK parameters were calculated using Kinetica analytical software.

#### *In vivo* PDX efficacy models

All *in vivo* studies were conducted by contract research with Charles River Labs Discovery Services (Freiburg, Germany). Tumor fragments for each model were implanted into the flank of multiple (N >6) 4–6 week-old female NMRI nude mice (Crl:NMRI-Foxn1nu). Mice with established tumors (80–200 mm^3^) were randomized and treated intravenously with either NAV-001-PNU on day 1, 8, and 15 post-randomization at 0.25 mg/kg or on day 1 at 0.25 or 0.75 mg/kg. Body weights and tumor volumes (mm^3^) were carried out by caliper measurement three times weekly. Termination of individual mice was carried out when tumor volume was ≥2,000 mm^3^ (unilateral tumors) or at a set study termination date.

#### Statistics

P values for data generated by ELISA were calculated using the Student’s T-test. All other P values were calculated via 2-Way ANOVA, with Dunnett’s multiple comparisons test. Statistical analyses were performed using GraphPad Prism 8.0 software. Results were considered to be significant if P < 0.05.

#### Ethics statement

All animal experiments have been approved by the internal Ethics Committee for Animal Experimentation at Charles River Labs and the Institutional Animal Care and Use Committee at Biocytogen. Mice were acclimatized for a week before starting the experiments. Mice were kept in individually ventilated filter top cages with free access to standard food and water ad libitum. All efforts were made to minimize suffering during experiments, including biweekly monitoring for weight loss and morbidity that was monitored by licensed veterinarians. Euthanasia of mice was carried out by providing animals a cocktail containing ketamine HCl (100 mg/mL) and xylazine HCl (100 mg/mL) intraperitoneally.

## Results and discussion

### Screening for MUC16/CA125 binding to anti-MSLN antibodies

**Fig 1:**
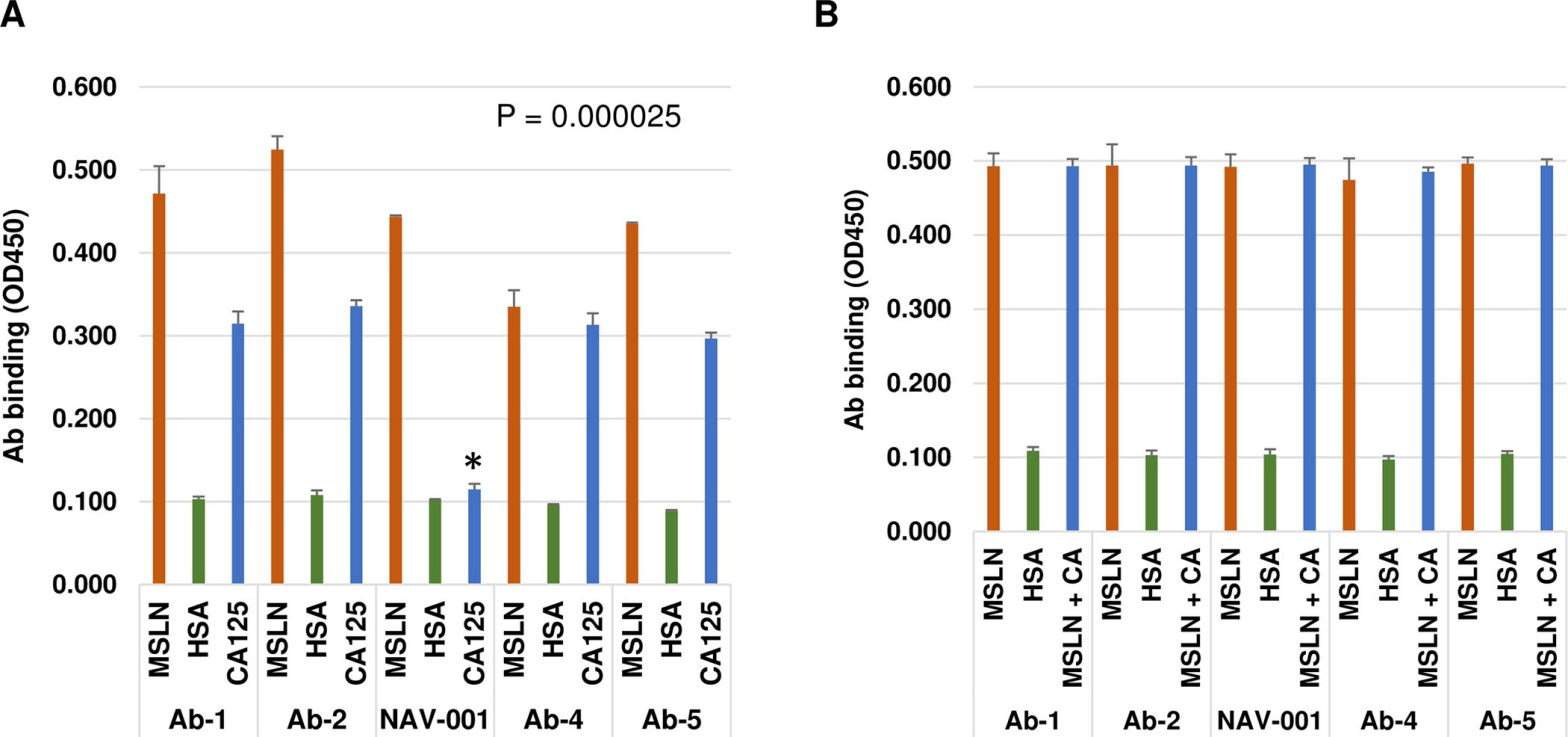
Screening anti-MSLN antibodies for binding to MUC16/CA125. Antibodies were screened via ELISA for binding to immobilized MSLN and MUC16/CA125 proteins. Human serum albumin (HSA) was used as negative control. Panel A shows that the antibody referred to herein as NAV-001 was the only antibody from the group that did not bind to MUC16/CA125 protein (P = 0.000025). To determine if MUC16/CA125 perturbs anti-MSLN binding to MSLN protein, immobilized MSLN was probed with each antibody with or without MUC16/CA125 (CA). As shown in panel B, MUC16/CA125 had no impact on antibodies binding to MSLN. All data represent a minimum of triplicate experiments.

Direct binding of anti-MSLN antibodies by MUC16/CA125 has been previously reported to suppress immune-effector activities and associated with decreased therapeutic activity in human clinical studies [2–5]. In an attempt to analyze as many existing anti-MSLN antibodies, we acquired rodent and human/humanized anti-MSLN antibodies from a variety of sources and ran preliminary screens for MUC16/CA125 binding. We narrowed down the preliminary screens to five antibodies that were humanized or fully human and retested them for binding to MSLN, MUC16/CA125 or HSA. As shown in Fig 1A, all antibodies recognized the MSLN antigen and 4 of 5 antibodies were also able to bind MUC16/CA125. We next tested if MUC16/CA125 can perturb anti-MSLN antibody binding to MSLN protein via competition assay. As shown in Fig 1B, MUC16/CA125 did not impact anti-MSLN binding to MSLN protein. NAV-001 was the only antibody tested that did not naturally bind to MUC16/CA125 (Fig 1A). NAV-001 has an affinity of 70 pM [13] and cross-reacts with cynomolgus monkey but not rodent MSLN ortholog proteins [S1 Fig]. This antibody and a subset of others were then reformatted into ADCs to test for the impact of MUC16/CA125 binding on ADC cytotoxicity.

### Effect of MUC16/CA125 binding on ADC cytotoxicity

**Fig 2:**
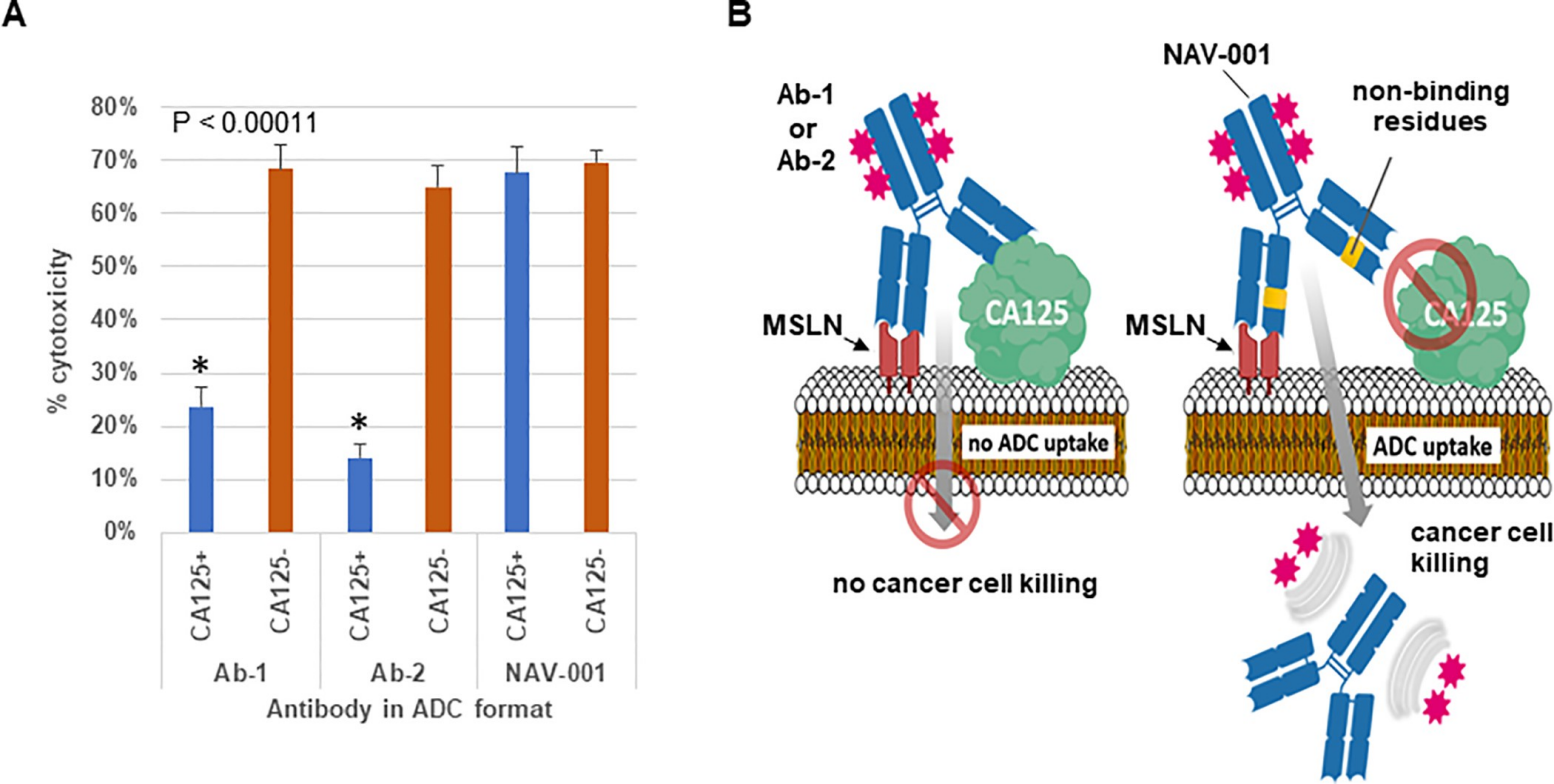
Testing MUC16/CA125 binding vs non-binding antibodies for cytotoxicity in ADC format. Antibodies Ab-1, Ab-2 and NAV-001 were converted into SN-38 ADCs and tested for cytotoxicity using the isogenic MSLN-expressing OVCAR-3 and OV-KD cells, the latter which lacks MUC16/CA125 expression. As shown in Panel A, Ab-1 and Ab-2 had reduced OVCAR-3 target cell (CA125^+^) killing as compared to NAV-001 (P < 0.00011). All three ADCs killed the isogenic OV-KD cells (CA125^-^) at a similar magnitude confirming the negative effect of MUC16/CA125 on ADCs containing antibodies that bind it. Shown is the differential killing of each ADC when cultures were treated with 50 ng/mL of each ADC. Panel B is a schematic representation of the potential mechanism by which MUC16/CA125 suppresses ADC killing through antibody binding and reduced internalization. All data represent a minimum of triplicate experiments.

Two of the MUC16/CA125 binding antibodies (Ab-1 and Ab-2) and the NAV-001 antibody were analyzed for killing activity against OVCAR-3 cells, which express both MSLN and MUC16/CA125, and the isogenic OV-KD MUC16/CA125 knockdown line. These lines express similar amounts of MSLN as determined by FACS and have similar morphology as well as growth properties. We first tested Ab-1, Ab-2 and NAV-001 antibodies as SN-38 ADCs using the CL2A PEG8-triazole-PABC-peptide-SN-38 linker-toxin as described in the methods. This linker is pH sensitive and is cleaved in lysosomes when internalized into target cells. SN-38 is a metabolite of irinotecan and a potent topoisomerase I inhibitor that has shown robust tumor cell killing that is dependent on internalization [14]. As shown in Fig 2A, the two antibodies bound by MUC16/CA125 (Ab-1 and Ab-2) had 2.8–4.9 fold less target cell killing of OVCAR-3 cells than the non-MUC16/CA125 binding NAV-001-SN-38, respectively. This effect appeared to be MUC16/CA125-dependent as the activity of all three ADCs had a similar potency against the MUC16/CA125 knockdown OV-KD cell line. As the extent of ADC-mediated cytotoxicity is affected by the degree of internalization, and because the difference between OVCAR-3 and OV-KD is the cell surface expression of MUC16/CA125, a potential mechanism for the different level of cytotoxicity may involve MUC16/CA125 binding, reduced internalization, and payload liberation. A schematic representation of this hypothesis is depicted in Fig 2B.

**Fig 3:**
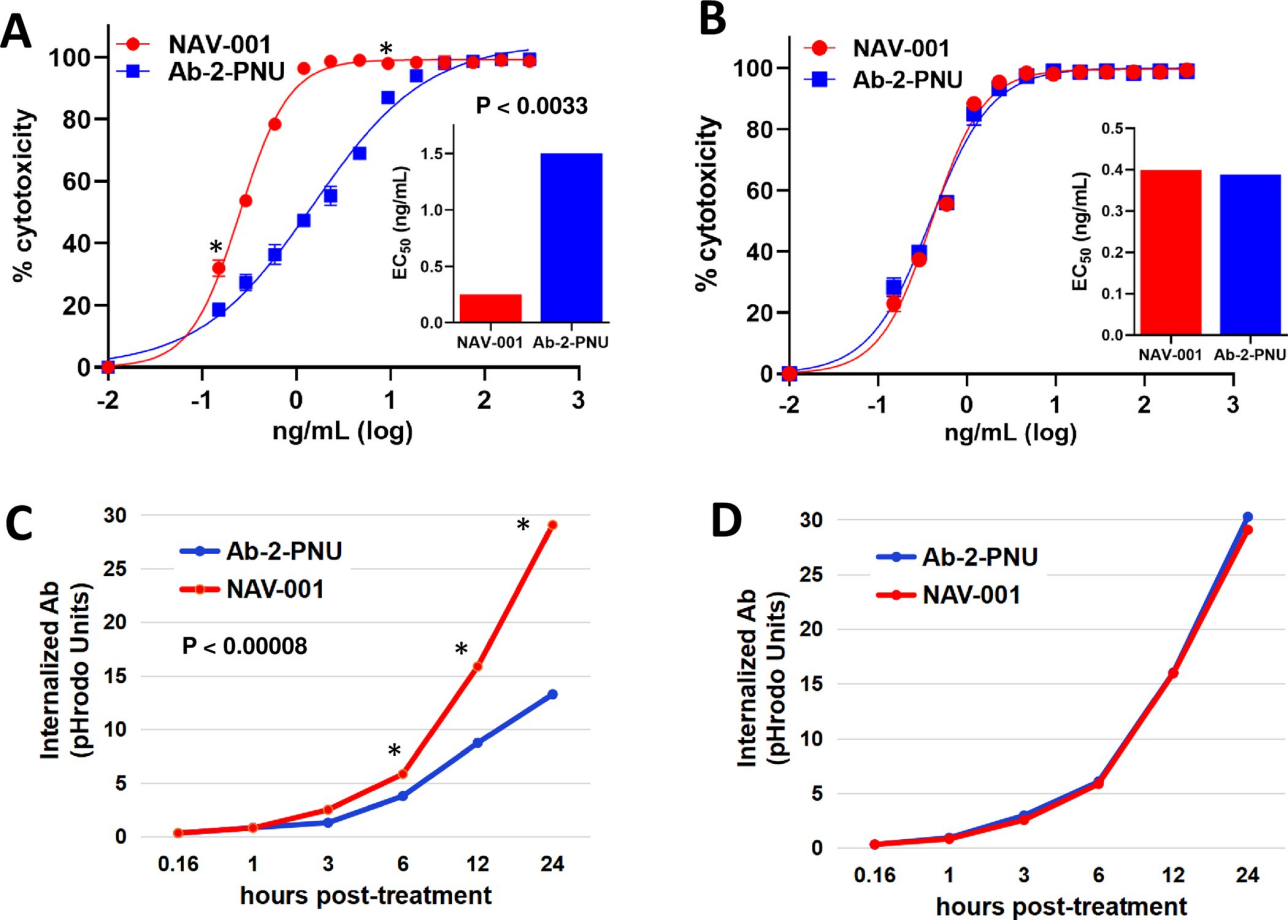
Retesting MUC16/CA125 binding vs non-binding antibodies for cytotoxicity in an alternative ADC format. Antibody 2 (Ab-2) and NAV-001 were converted into ADCs by linking the topoisomerase II cytotoxin PNU-159682 and both were tested for cytotoxicity against the isogenic MSLN-expressing OVCAR-3 (panel A and C) and OV-KD (panel B and D) cell lines. As shown in Panels A and B, a similar effect of target cell killing was observed as the SN-38 formatted ADCs shown in Fig 2A, whereby the MUC16/CA125 binding Ab-2-PNU had reduced killing in OVCAR-3 (A) as compared to NAV-001-PNU(P < 0.0033), while similar killing was observed for both against the isogenic non-MUC16/CA125-expressing OV-KD cells (B). Antibodies were tested for cellular uptake using the same cell pairs and as shown in Panels C (OVCAR-3) and D (OV-KD), Ab-2 has reduced internalization as compared to NAV-001 in OVCAR-3 cells (P < 0.00008) but similar internalization kinetics in the OV-KD cells. All data represent a minimum of triplicate experiments.

To confirm the cytotoxicity activities observed with the SN-38 ADCs, we reformatted the Ab-2 and NAV-001 antibodies to a different payload using a different linker (see below). Here the antibodies were linked to the nemorubicin metabolite, PNU-159682 (referred to as PNU). PNU has been shown to be a strong topoisomerase II inhibitor, effective against auristatin and maytansinoid resistant tumors and a non-P-pg substrate, making it refractory to multi-drug resistance (MDR) mechanisms [15]. The Ab-2-PNU was found to have a DAR of 3.99 while the NAV-001-PNU had a DAR of 3.45. Binding of each ADC to MSLN and MUC16/CA125 were similar to that observed in the unconjugated formats shown in Fig 1A. Both NAV-001-PNU and Ab-2-PNU were next tested for cytotoxicity against the isogenic OVCAR-3 and OV-KD cell lines. As shown in Fig 3A, NAV-001-PNU ADC had a 6-fold higher killing of OVCAR-3 than Ab-2-PNU (EC_50_ 0.25 vs 1.25 ng/mL, respectively). As found using the SN-38 ADCs, similar levels of killing were observed for both PNU ADCs against the OV-KD MUC16/CA125 knockdown cells (EC_50_ 0.39 vs 0.38 ng/mL, respectively) (Fig 3B).

### Internalization of NAV-001 and Ab-2 antibodies in OVCAR-3 and OV-KD cells

To determine if the differential killing observed between MUC16/CA125 bound vs non-bound ADCs were due to altered internalization rates, the PHrodo™ Red Avidin internalization diagnostic was used to monitor NAV-001 and Ab-2 uptake by OVCAR-3 and OV-KD cells over a 24-hour period [16]. As shown in Fig 3C, NAV-001 antibody had a faster internalization rate than Ab-2 in OVCAR-3 cells, while both antibodies showed similar internalization rates in the OV-KD MUC16/CA125 knockdown cells (Fig 3D). No internalization was observed for the non-biotinylated NAV-001 or Ab-2 as expected since neither contained PHrodo Red fluor. A 10-fold addition of non-labeled antibodies to PHrodo Red labeled antibodies suppressed internalization, further demonstrating specificity of MSLN-mediated uptake.

### NAV-001-PNU design and bystander killing activity

**Fig 4:**
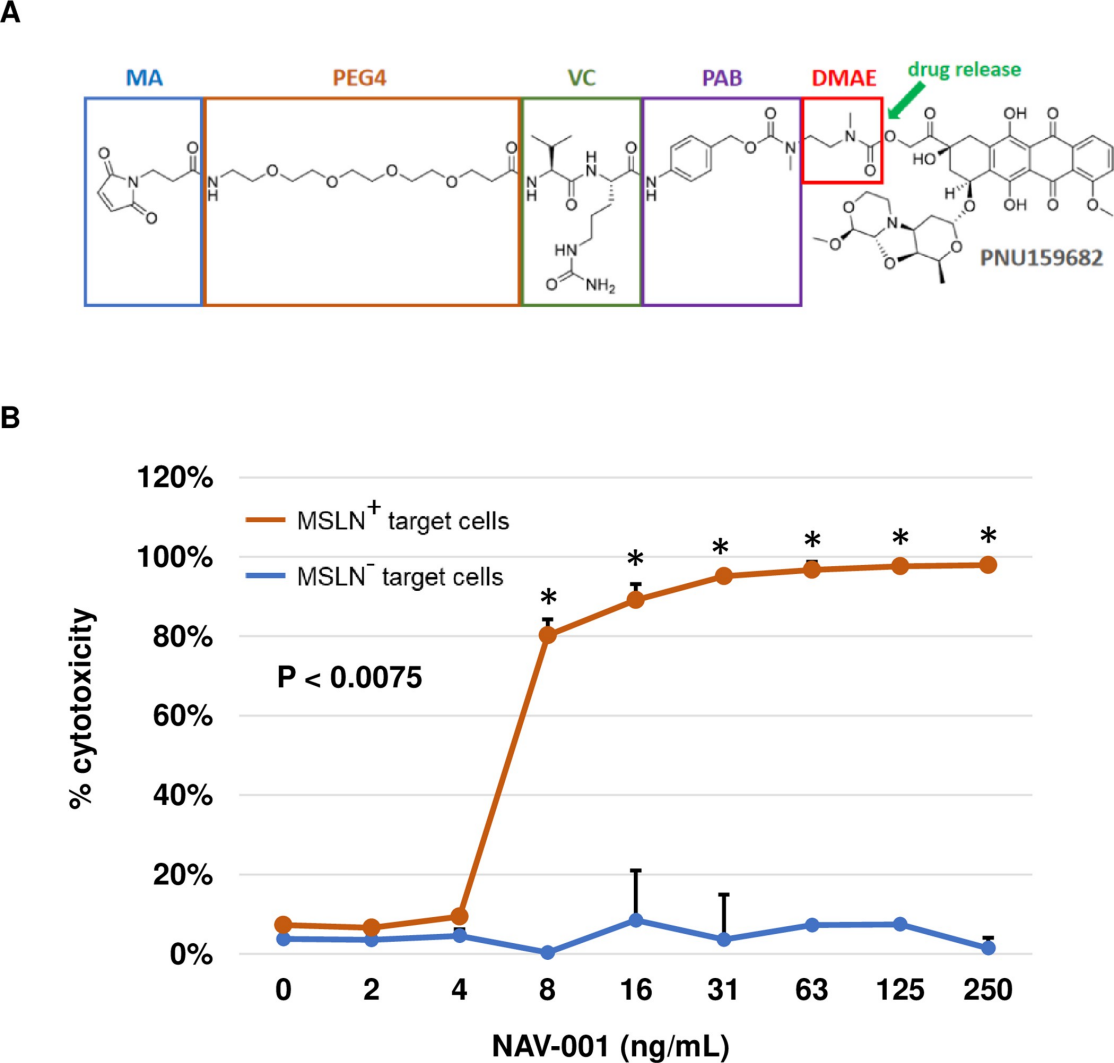
NAV-001-PNU has robust bystander cell killing activity. Panel A, structure of NAV-001-PNU’s linker and cytotoxic payload comprising the following groups: N-maleoyl-B-alanine (MA) as the end group for attachment to antibody (humanized IgG_1_) via reduced interchain Cys-maleimide chemistry; polyethylene glycol (PEG4) to increase ADC solubility; cathepsin B protease-sensitive valine-citrulline (VC) dipeptide; self-immolative spacer p-aminobenzylcarbamate (PAB) and dimethylaminoethanol (DMAE) for the attachment and release of the intact PNU-159682 cytotoxin. Panel B, MSLN-negative Jurkat cells were treated with NAV-001-PNU, and either co-cocultured with MSLN-positive NCI-N87 cells (red line) or cultured alone (blue line). Jurkat cytotoxicity was observed only when cells were co-cultured with NCI-N87 cells (P < 0.0075), indicating a requirement for NAV-001-PNU processing followed by PNU-159682 liberation and diffusion into the microculture environment after ADC uptake by MSLN-positive target cells. All data represent a minimum of triplicate experiments.

Many tumors have shown heterogeneous expression of target antigens, including MSLN-positive cancers, that has hampered the goal of total tumor cell eradication. Targeted delivery of payloads to tumors and their microenvironments offers the ability to deploy localized toxins that may kill target expressing and non-target expressing cells within the tumor microenvironment. ADCs that exhibit bystander cell killing, whereby liberated cytotoxic payload within target antigen-expressing cells is leaked out into the tumor microenvironment and is able to kill target-negative resident cells, have been shown to be more efficacious than those lacking this effect [17]. To increase the potential bystander activity of NAV-001-PNU, we employed the MA-PEG4-VC-PAB-DMAE linker that consists of the self-immolative spacer p-aminobenzylcarbamate (PAB) linked to dimethylaminoethanol (DMAE) for the attachment and release of intact PNU-159682 without any additional linker groups (Fig 4A), thereby enabling it to effectively diffuse out of internalized MSLN-expressing target cells and potentially kill MSLN-negative resident tumor and non-tumor cells within the microenvironment. The linker also contains a cathepsin B protease-sensitive valine-citrulline dipeptide (VC) for effective intracellular cleavage upon internalization.

To determine NAV-001-PNU bystander activity against cells with low or no MSLN expression, a co-culture system was employed testing NAV-001-PNU cytotoxicity in the presence or absence of MSLN-expressing target cells. Non-adherent, MSLN-negative Jurkat cells were cultured in the presence of 1–500 ng/mL of NAV-001-PNU or 0.1–100 pM of free PNU-159682 in wells seeded with the adherent MSLN-positive NCI-N87 gastric carcinoma line, adherent MSLN-negative CHO cells or no cells. While the increased ratio of ADC target cells to non-target cells has been shown to influence *in vitro* bystander killing [18], we chose to use a more conservative 1:1 ratio of cells. This ratio was based upon previous bystander assays that we have carried out while testing other ADCs. Briefly, wells were harvested at 96 hours and both Jurkat and adherent ADC target cells were tested for viability as described in the methods. As shown in Fig 4B, the EC_50_ of NAV-001-PNU processed by the NCI-N87 target cells on Jurkat cells was 12 ng/mL (equivalent to 79.1 pM), while the EC_50_ of NAV-001-PNU not processed by MSLN-negative CHO control cells on Jurkat cells was >750 ng/mL. These data demonstrate a robust bystander effect of NAV-001-PNU to maximize the potential cytotoxicity of tumors with heterogeneous MSLN-expression as well as other resident cell types in the tumor microenvironment.

### NAV-001 serum stability and pharmacokinetic analysis in mice

**Fig 5:**
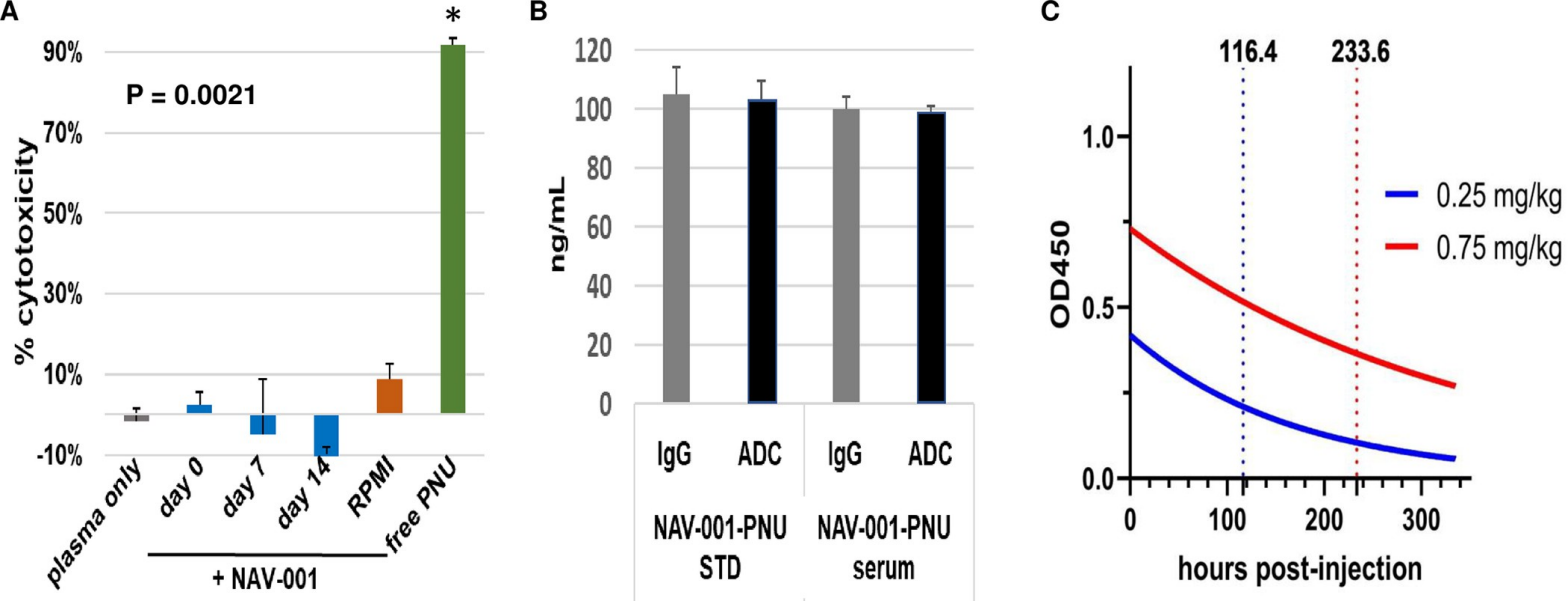
Serum stability and pharmacokinetics. Panel A is a bioassay measuring the liberation of the PNU-159682 (PNU) cytotoxin on MSLN-negative A549 cells after 14 days of incubation at 37°C in cynomolgus monkey plasma. NAV-001-PNU toxicity would be observed if >1% of the PNU was liberated from the ADC. As shown, NAV-001-PNU remains intact for >14 days in serum (P = 0.0021). Panel B is an ELISA confirming the bioassay data that NAV-001-PNU remains intact after 14 days of incubation in host plasma at 37°C. Samples were diluted to the theoretical input amounts in serum (50 ng/mL) and tested in ELISA for total IgG and intact ADC that showed no difference between IgG and ADC concentrations after serum incubation as observed in untreated controls (STD). Panel C shows the pharmacokinetic profile of a single-dose of 0.25 or 0.75 mg/kg NAV-001 administered to naïve CD1 mice via ELISA assay using anti-PNU capture and anti-IgG-HRP detector antibodies. All data represent a minimum of triplicate experiments.

Long-term stability of ADCs in systemic circulation is an important feature to minimize toxicity due to premature liberation of cytotoxic payload. To determine NAV-001-PNU stability, we assayed its stability in plasma from various species via bioassay that measures free PNU-159682 and ELISA that can detect the total antibody component as well as intact ADC. Bioassays were established as described in the methods and stressed serum samples were tested for cytotoxicity against MSLN-negative A549 cells. As shown in Fig 5A, NAV-001-PNU incubated with cynomolgus monkey plasma for 14 days at 37°C did not result in any significant liberation of free payload as measured by the low to no cytotoxicity against MSLN-negative cells, suggesting stability of NAV-001 under these conditions. Similar stability was observed using human, rat and hamster plasma (S2A Fig). The serum carboxylesterase 1C in purified mouse plasma has been reported to cause liberation of linker-payload for other ADCs [19] and served as a positive control for the sensitivity of this assay to liberate PNU-159682 while heat-inactivation of the plasma reduced its activity (S2A Fig). To further confirm NAV-001-PNU stability, ELISA analysis was performed on the stressed samples measuring the total antibody and intact ADC. Samples were diluted to a calculated amount of 50 ng/mL of NAV-001-PNU that should be contained within the stressed samples and quantified using a NAV-001-PNU standard. As shown in Fig 5B, concentration of total antibody and intact ADC were equivalent to the theoretical amount within the stressed plasma sample, therefore corroborating the bioassay findings. In addition, NAV-001-PNU is stable for up to 14 days in human serum (S2B Fig). These results demonstrate that NAV-001-PNU is highly stable in the serum of species being targeted for therapy and GLP toxicology studies.

Mouse PDX models are considered an accepted means to predictively evaluate the efficacy and safety of experimental agents [20]. Pharmacokinetic analysis of NAV-001-PNU was conducted in mice to determine its *in vivo* half-life and stability at dose levels found to be efficacious in pilot studies. Naïve CD-1 mice were administered 0.25 or 0.75 mg/kg of NAV-001-PNU intravenously and plasma was collected from treated mice at various timepoints and quantified by ELISA for total antibody and intact ADC using a noncompartmental analysis. As shown in Fig 5C, total antibody and ADC concentration curves appeared similar for both doses with the T_1/2_ being 4.8 days in 0.25 mg/kg and 9.7 days in 0.75 mg/kg dosed animals. No toxicity was observed in any mice. These data demonstrate that NAV-001-PNU ADC is stable in plasma *in vitro* and *in vivo*.

### NAV-001-PNU *in vivo* efficacy in PDX models

**Fig 6:**
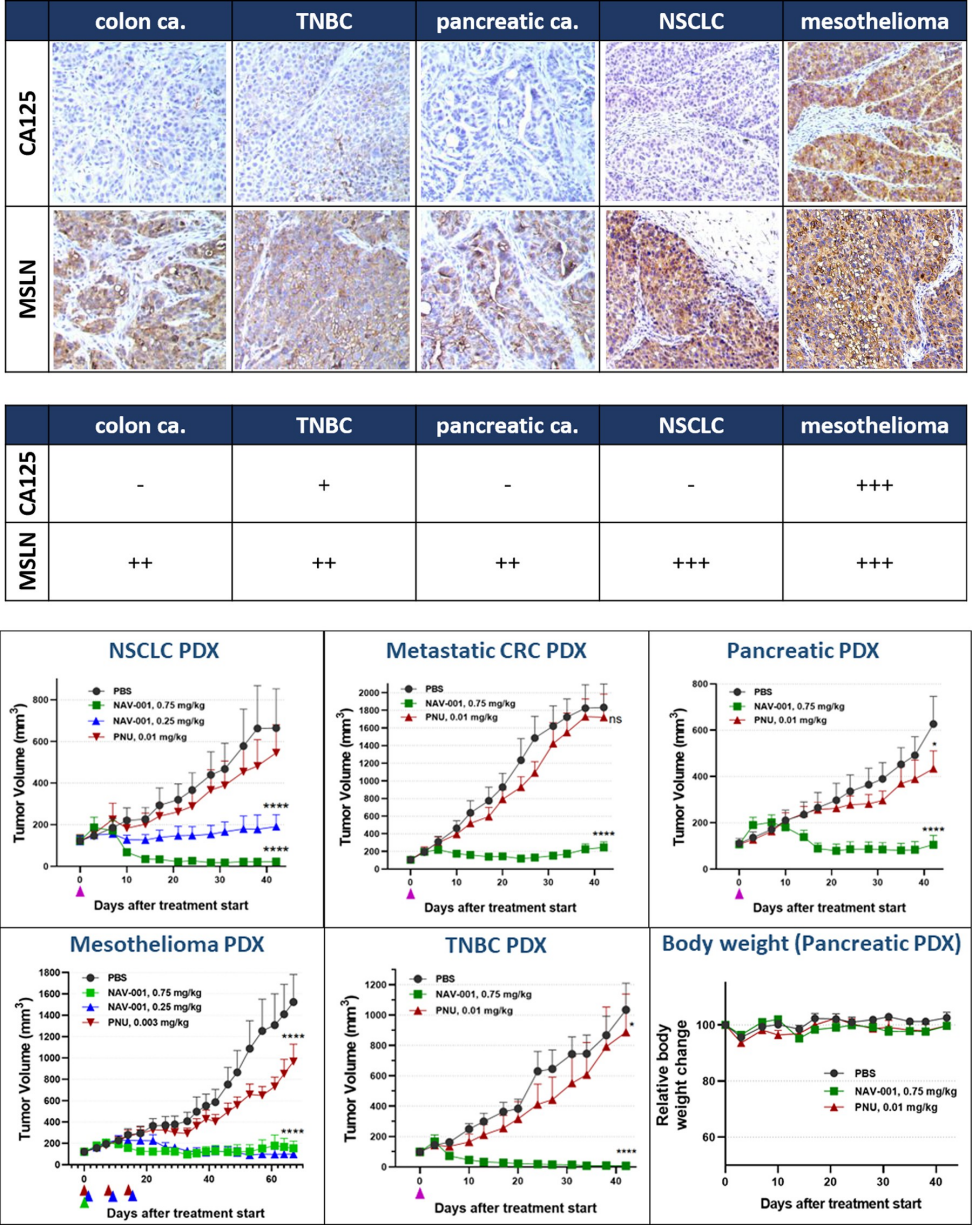
*In vivo* efficacy and tolerability of NAV-001-PNU in MUC16/CA125 positive and negative PDX models. Panel 6A, PDFs used for each study were confirmed for MSLN and MUC16/CA125 expression by IHC analyses. As shown, all tumors had robust homogeneous MSLN staining, while the mesothelioma and TNBC tumors also expressed MUC16/CA125. These tumors served as models to confirm NAV-001-PNU *in vivo* efficacy against MUC16/CA125 expressing tumors. Panel B, tumor growth and tolerability of NAV-001 in PDX models. As shown, single-dose of NAV-001 at 0.25 or 0.75 mg/kg showed significant anti-tumor efficacy (P < 0.005), and long-term regression after 0.75 mg/kg single-dose regimen. Triangles represent day of dosing post randomization. Each cohort of each study employed 6 mice.

To evaluate the therapeutic efficacy of NAV-001-PNU ADC, we first screened PDFs for MSLN and MUC16/CA125 expression by *in silico* RNA analysis and then confirmed expression via IHC. As shown in Fig 6A, MSLN expression was confirmed in five PDFs: CXF533 metastatic colorectal cancer (CRC), MAXFTN574 triple negative breast cancer (TNBC), PAXF2057 pancreatic cancer, LXFA983 metastatic non-small cell lung adenocarcinoma (NSCLC) and PXF1118 malignant pleural mesothelioma (MPM). Of the five tumors, two (TNBC—moderate expression; MPM—high expression) were found to also express MUC16/CA125. We first tested NAV-001-PNU activity in MPM (high MUC16/CA125) and NSCLC (no/low MUC16/CA125) PDX models. Athymic nude mice with 80–200 mm^3^ tumors were randomized and treated intravenously with NAV-001-PNU either once a week for three weeks at 0.25 mg/kg or once at 0.25 or 0.75 mg/kg. Free PNU-159682 was administered once a week for three weeks at levels equimolar to the amount conjugated to NAV-001-PNU dosed at 0.75 mg/kg. PBS treated mice served as controls. As shown in Fig 6B, in PBS and PNU control groups tumors more than doubled at day 20 after randomization, while mice treated with NAV-001-PNU showed durable (≥42 days) tumor growth suppression when treated with a single dose at 0.25 mg/kg in NSCLC PDX or complete tumor regression when treated with repeat dosing at 0.25 mg/kg or single dosing at 0.75 mg/kg. Mice treated with free PNU-159682 showed slight tumor growth delay as compared to PBS treated mice. Since single-dose NAV-001-PNU at 0.75 mg/kg showed durable responses in both the NSCLC and MPM models, we employed this dosing scheme to determine activity against other MSLN-expressing PDX models. As shown, single-dose NAV-001-PNU had robust efficacy in CRC, pancreatic and TNBC PDX models, the latter of which was mildly positive for MUC16/CA125. NAV-001-PNU was well tolerated in all treatment regimens as determined by absence of weight loss and lack of symptomatic observations. A representative analysis of body weights is shown in the right lower panel in Fig 6B. In total, these data demonstrate NAV-001-PNU *in vivo* potency across a spectrum of MSLN-expressing tumors in models where sub-mg/kg single dose treatment is sufficient for complete tumor regression and long-term durable response regardless of MUC16/CA125 status.

With the recent clinical success of ADCs in a subset of unmet cancer indications, a concerted effort continues to be employed across the industry to improve upon their therapeutic potency. Much of this research is focused on improving linker-drug stability, cytotoxin potency, enhanced IgG_1_ antibody affinity and Fc optimization while the pursuit of less obvious factors that may negatively impact ADC therapeutic activity remains overlooked [21]. We and others have previously found that subsets of HIO factors (i.e., MUC16/CA125) bind to IgG type immunoglobulins [2–5, 12, 22]. Moreover, this binding has been shown to have profound negative effects on IgG_1_ immune-effector activities that diminishes their therapeutic potential [1, 2, 4]. Based on these observations, we hypothesized that engineering HIO-affected antibodies in new formats such as ADCs or CAR-Ts may enable them to overcome such suppression. To test this hypothesis, we designed ADCs that are susceptible or refractory to MUC16/CA125 HIO factor binding. Assays comparing their cytotoxicity against isogenic cell lines that were MUC16/CA125 positive or negative were quantified using methods confirmed to accurately measure cell growth kinetics and viability [23]. Our studies found that HIO factors can negatively impact alternative antibody-based formats such as ADCs similar to their parent IgG (Figs 2A and 3B). This effect appears to involve reduced ADC cellular uptake via binding of the ADC’s antibody component to cell surface MUC16/CA125 (Fig 3C and 3D). The rate of ADC uptake is a feature known to be important for ADC therapeutic activity [24]. Fig 2B provides a schematic representation of the proposed suppressive mechanism that is supported by the cytotoxic observations and cellular uptake results shown in Fig 3. Importantly, these findings suggest that a broader list of parameters should be considered when designing ADC-based agents for cancer therapy.

MSLN is viewed as a widely validated therapeutic target in cancer therapy due to its over-expression in many cancer types [25]. Several anti-MSLN experimental therapies are currently being pursued that are formatted as canonical naked IgG_1_ antibodies that rely on immune-effector cell killing, bispecific antibodies, single chain immunotoxins, ADCs and CAR-Ts. While the development of anti-MSLN therapies continues to remain active in clinical cancer research, its success for effective targeting and therapeutic outcomes has been challenging for a multitude of reasons. A Phase 2 clinical trial of the IgG_1_ amatuximab found it to be mildly effective in patients with stage III/IV primary nonresectable malignant pleural mesothelioma (MPM) [26]. *Post hoc* analysis of the study found that patients with low MUC16/CA125 levels had a 2- and 7-month improvement in progression free survival (HR 0.43, *P* = 0.0062) and overall survival (HR 0.40, *P* = 0.0022) respectively, in contrast to those with elevated MUC16/CA125 levels [4]. Translational evidence found that amatuximab is susceptible to MUC16/CA125 binding and suppressed immune-effector activity [4]. These data suggest a link between amatuximab therapeutic efficacy in patients with baseline vs. elevated MUC16/CA125 levels, which is a variable that may affect its clinical utility. SS1P is an immunotoxin containing a single chain anti-MSLN targeting antibody genetically fused to the Pseudomonas exotoxin A. It has been tested in several clinical trials but has had limited success due to the immunogenicity of the exotoxin component [27]. Several bispecific antibody formats that target tumor-expressed MSLN and receptors on T cells (i.e., CD3, CD40, etc.) have been developed in attempts to better juxtapose cytotoxic T-cells to the tumor’s cell surface to improve anti-tumor immune responses [28]. While some of these agents have shown robust preclinical activity, undefined immune-mediated toxicities and/or lack of efficacy has limited their development in human trials and more refinement is required [29].

The use of MSLN-targeted ADCs has been pursued by several groups. To date, three anti-MSLN ADCs have been tested in clinical trials. Two employ anti-mitotic cytotoxins (anetumab ravtansine and DMOT4039A), and the third employs the DNA alkylating agent duocarmycin (BMS-986148) [30]. While these ADCs have shown good preclinical efficacy and tolerability in clinical trials, their clinical efficacy has been modest or ineffective [31–33]. Interestingly, all clinical trials have involved cancers that are associated with elevated MUC16/CA125. In an attempt to evaluate these ADCs for HIO factor susceptibility, we were able to purchase the MF-T antibody (anetumab) and its ADC (MF-T-DM4) (Creative Biolabs) [32]. ELISA analysis of MF-T found that it was bound by MUC16/CA125 (S3A Fig) and *in vitro* cytotoxicity analysis of its ADC format found that it was approximately 3 logs less potent than NAV-001-PNU when tested head-to-head (S3B Fig). The authors of a clinical trial testing the DOT4039A ADC observed that tumor responses were much lower in pancreatic patients, a cancer in which >90% of patients over-express MUC16/CA125 [8]. They cited that ADC tumor uptake could be a potential mechanism of resistance [33]. This was supported by the finding of a Phase 1 imaging study showing that radio-labeled amatuximab had higher uptake in mesothelioma tumors than pancreatic tumors, the latter of which express higher levels of MUC16/CA125 [34].

Finally, the impact of CAR-Ts employing the use of antibody sequences that may be susceptible to HIO factor binding is unknown. A recent study using anti-MSLN CAR-T cells consisting of the SS1 antibody and a construct consisting of antibody sequences composing NAV-001 showed inherent differences *in vivo* against multiple MSLN and MUC16/CA125 expressing tumor cell lines [13]. Their data found that NAV-001 CAR-Ts were more effective than SS1 CAR-Ts. The authors commented that the epitopes for the two antibodies are on opposite ends of the protein and may be a cause for better NAV-001 tumor penetration and tumor cell killing. Alternatively, HIO factor binding to the antibody sequences within the SS1 CAR-T could be another potential reason for its reduced tumor penetration and killing. Lack of tumor penetration was also hypothesized using a similar SS1 CAR-T in a human Phase 1 clinical trial that showed modest tumor killing [35].

The above data suggest that HIO factor binding to antibody-mediated cancer therapies can potentially impact their therapeutic activity. Consideration and empirical testing of HIO factor binding using methods described herein may be useful in designing alternative antibody-based formats such as ADCs in early stage development.

## Conclusion

Significant efforts to improve the safety and efficacy of ADC-based therapeutics continue to be actively pursued across the biopharmaceutical industry. Here, we show that HIO factors that bind to ADCs can have a significant impact on target cell killing. These data suggest that screening ADC candidates for HIO factor sensitivity should be another parameter to consider during their development in addition to potency, stability and bystander activity. Development strategies similar to those used to generate NAV-001-PNU may potentially lead to improved therapeutic responses in patients with cancers overexpressing HIO factors such as MUC16/CA125.

## Supporting information

S1 Fig NAV-001 cross-reacts with cynomolgus monkey but not rat MSLN ortholog proteins. MSLN ortholog proteins (Sino Biologicals) were dot blotted in duplicate (100 ng/dot) on PVDF membrane, blocked with PBS-0.5% Tween 20 (PBS-T) plus 5% dry milk for 1 hour, and probed with 1 μg/mL NAV-001 or a negative control antibody (PTZ) for 1 hour. Membranes were washed with PBS-T and bound antibodies were detected using 40 ng/mL of the anti-human IgG-HRP (Jackson ImmunoResearch) for 20 minutes, washed and detected by enhanced chemiluminescence (ECL) substrate (SuperSignal, ThermoFisher). Signals were quantified by densitometry using the iBright software version 5. No difference in NAV-001 binding was observed between cynomolgus monkey (cyno, blue bar) or human (hum, red bar) MSLN (P = 0.37) while no binding was observed to the rat MSLN.
(PDF)

S2 Fig NAV-001 is stable in human, rat and hamster plasma. Bioassays were employed to measure the liberation of PNU-159682 cytotoxin (PNU) from NAV-001-PNU (NAV-001) and its cytotoxic effect on MSLN-negative A549 cells after incubation of NAV-001 at 37°C for 7 days in rat, cynomolgus monkey (cyno), hamster (panel A) or human (panel B) plasma. The temperature-sensitive serum carboxylesterase 1C naturally found in purified mouse plasma (panel A, right brown bar), and is known to cleave linkers containing chemical motifs included in the NAV-001 linker-toxin [19], served as a positive control for the sensitivity of this assay to liberated PNU. The enzyme activity could be neutralized by heat-inactivation of mouse plasma for 20 minutes at 60°C (panel A, left brown bar). For human plasma studies, free PNU served as a positive control to monitor assay sensitivity (panel B, green bar). Both controls were statistically significant when compared to the stability of NAV-001 in human, cyno, rat and hamster plasmas (P < 0.016).
(PDF)

S3 Fig Comparative MUC16/CA125 binding and target cell killing of MF-T-DM4 (anetumab ravtansine) and NAV-001-PNU. ELISA antibody-CA125 binding assays showed that the MF-T antibody (anetumab) was significantly bound by MUC16/CA125 in contrast to NAV-001 (panel A) (P < 0.00002) and was less effective in killing MSLN-expressing NCI- N87 target cells than NAV-001-PNU when in ADC format (anetumab ravtansine) (panel B). All data represent a minimum of triplicate experiments.
(PDF)
