# Supplementary material for: NAV-001, a high-efficacy antibody-drug conjugate targeting mesothelin with improved delivery of a potent payload by counteracting MUC16/CA125 inhibitory effects
Source: PLoS One. 2023 May 17;18(5):e0285161. doi: 10.1371/journal.pone.0285161 (PMC10191272; doi:10.1371/journal.pone.0285161)

S1 Fig. NAV-001 cross-reacts with cynomolgus monkey but not rat MSLN ortholog proteins. MSLN ortholog proteins (Sino Biologicals) were dot blotted in duplicate (100 ng/dot) on PVDF membrane, blocked with PBS-0.5% Tween 20 (PBS-T) plus 5% dry milk for 1 hour, and probed with 1 µg/mL NAV-001 or a negative control antibody (PTZ) for 1 hour. Membranes were washed with PBS-T and bound antibodies were detected using 40 ng/mL of the anti-human IgG-HRP (Jackson ImmunoResearch) for 20 minutes, washed and detected by enhanced chemiluminescence (ECL) substrate (SuperSignal, ThermoFisher). Signals were quantified by densitometry using the iBright software version 5. No difference in NAV-001 binding was observed between cynomolgus monkey (cyno, blue bar) or human (hum, red bar) MSLN (P = 0.37) while no binding was observed to the rat MSLN.

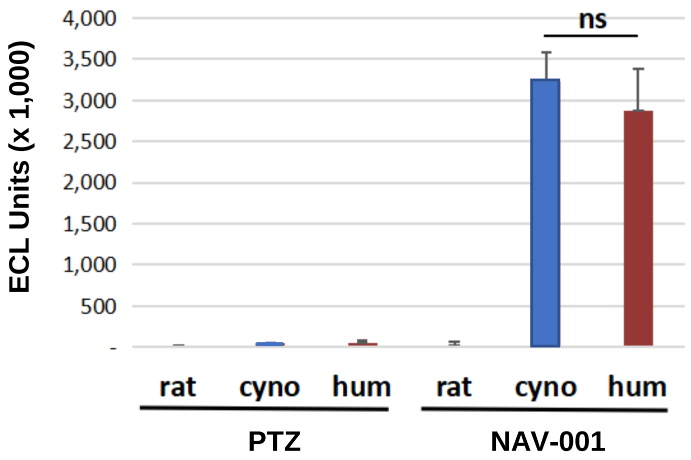

Supplement: S1 Fig — MSLN ortholog proteins (Sino Biologicals) were dot blotted in duplicate (100 ng/dot) on PVDF membrane, blocked with PBS-0.5% Tween 20 (PBS-T) plus 5% dry milk for 1 hour, and probed with 1 μg/mL NAV-001 or a negative control antibody (PTZ) for 1 hour. Membranes were washed with PBS-T and bound antibodies were detected using 40 ng/mL of the anti-human IgG-HRP (Jackson ImmunoResearch) for 20 minutes, washed and detected by enhanced chemiluminescence (ECL) substrate (SuperSignal, ThermoFisher). Signals were quantified by densitometry using the iBright software version 5. No difference in NAV-001 binding was observed between cynomolgus monkey (cyno, blue bar) or human (hum, red bar) MSLN (P = 0.37) while no binding was observed to the rat MSLN. (PDF) [file pone.0285161.s001.pdf]
