# Supplementary material for: NAV-001, a high-efficacy antibody-drug conjugate targeting mesothelin with improved delivery of a potent payload by counteracting MUC16/CA125 inhibitory effects
Source: PLoS One. 2023 May 17;18(5):e0285161. doi: 10.1371/journal.pone.0285161 (PMC10191272; doi:10.1371/journal.pone.0285161)

S2 Fig. NAV-001 is stable in human, cynomolgus monkey, rat and hamster plasma. Bioassays were employed to measure the liberation of PNU-159682 cytotoxin (PNU) from NAV-001-PNU (NAV-001) and its cytotoxic effect on MSLN-negative A549 cells after incubation of NAV-001 at 37°C for 7 days in rat, cynomolgus monkey (cyno), hamster (panel A) or human (panel B) plasma. The temperature-sensitive serum carboxylesterase 1C naturally found in purified mouse plasma (panel A, right brown bar), and is known to cleave linkers containing chemical motifs included in the NAV-001 linker-toxin [19], served as a positive control for the sensitivity of this assay to liberated PNU. The enzyme activity could be neutralized by heat-inactivation of mouse plasma for 20 minutes at 60°C (panel A, left brown bar). For human plasma studies, free PNU served as a positive control to monitor assay sensitivity (panel B, green bar). Both controls were statistically significant when compared to the stability of NAV-001 in human, cyno, rat and hamster plasmas (P < 0.016).

A

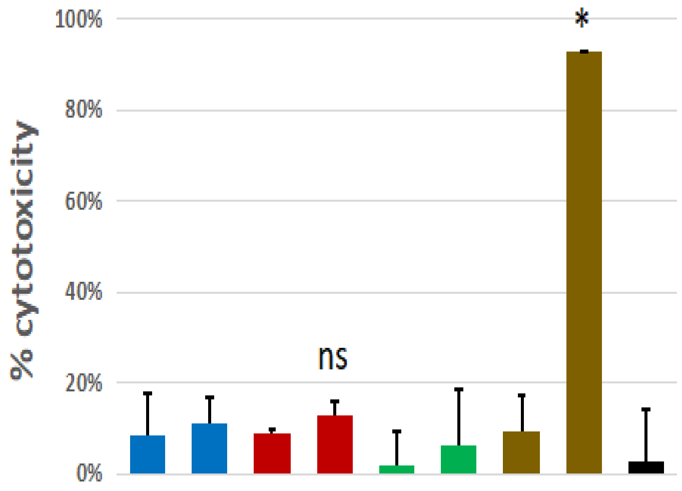

plasma	rat		cyno		ham		mouse		RPMI
37°C, days	0	7	0	7	0	7			
37°C, hours							1	1	0
NAV-001 ng/mL	100	100	100	100	100	100	100	100	100
heat inactivation	-	-	-	-	-	-	+	-	-

B

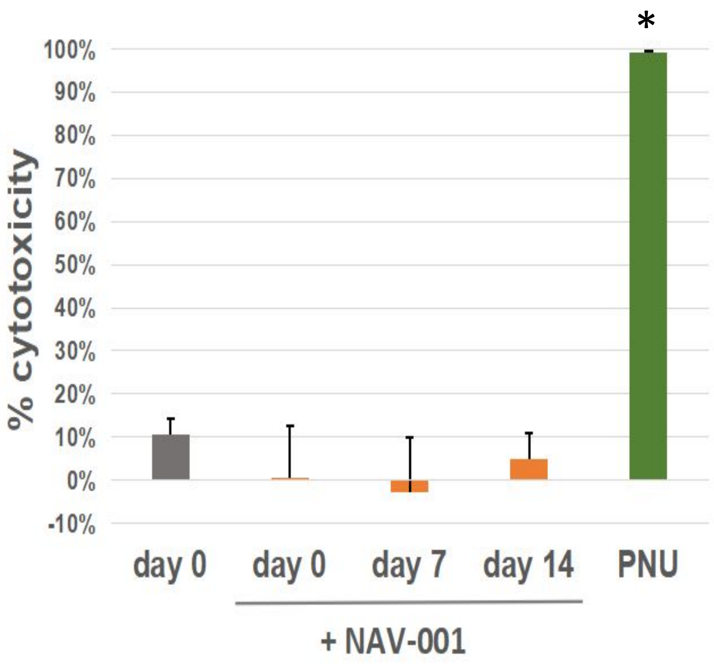

Supplement: S2 Fig — Bioassays were employed to measure the liberation of PNU-159682 cytotoxin (PNU) from NAV-001-PNU (NAV-001) and its cytotoxic effect on MSLN-negative A549 cells after incubation of NAV-001 at 37°C for 7 days in rat, cynomolgus monkey (cyno), hamster (panel A) or human (panel B) plasma. The temperature-sensitive serum carboxylesterase 1C naturally found in purified mouse plasma (panel A, right brown bar), and is known to cleave linkers containing chemical motifs included in the NAV-001 linker-toxin [19], served as a positive control for the sensitivity of this assay to liberated PNU. The enzyme activity could be neutralized by heat-inactivation of mouse plasma for 20 minutes at 60°C (panel A, left brown bar). For human plasma studies, free PNU served as a positive control to monitor assay sensitivity (panel B, green bar). Both controls were statistically significant when compared to the stability of NAV-001 in human, cyno, rat and hamster plasmas (P < 0.016). (PDF) [file pone.0285161.s002.pdf]
