# Supplementary material for: NAV-001, a high-efficacy antibody-drug conjugate targeting mesothelin with improved delivery of a potent payload by counteracting MUC16/CA125 inhibitory effects
Source: PLoS One. 2023 May 17;18(5):e0285161. doi: 10.1371/journal.pone.0285161 (PMC10191272; doi:10.1371/journal.pone.0285161)

S3 Fig. Comparative MUC16/CA125 binding and target cell killing of MF-T-DM4 (anetumab ravtansine) and NAV-001-PNU. ELISA antibody-CA125 binding assays showed that the MF-T antibody (anetumab) was significantly bound by MUC16/CA125 in contrast to NAV-001 (panel A) ($P < 0.00002$) and was less effective in killing MSLN-expressing NCI-N87 target cells than NAV-001-PNU when in ADC format (anetumab ravtansine) (panel B). All data represent a minimum of triplicate experiments.

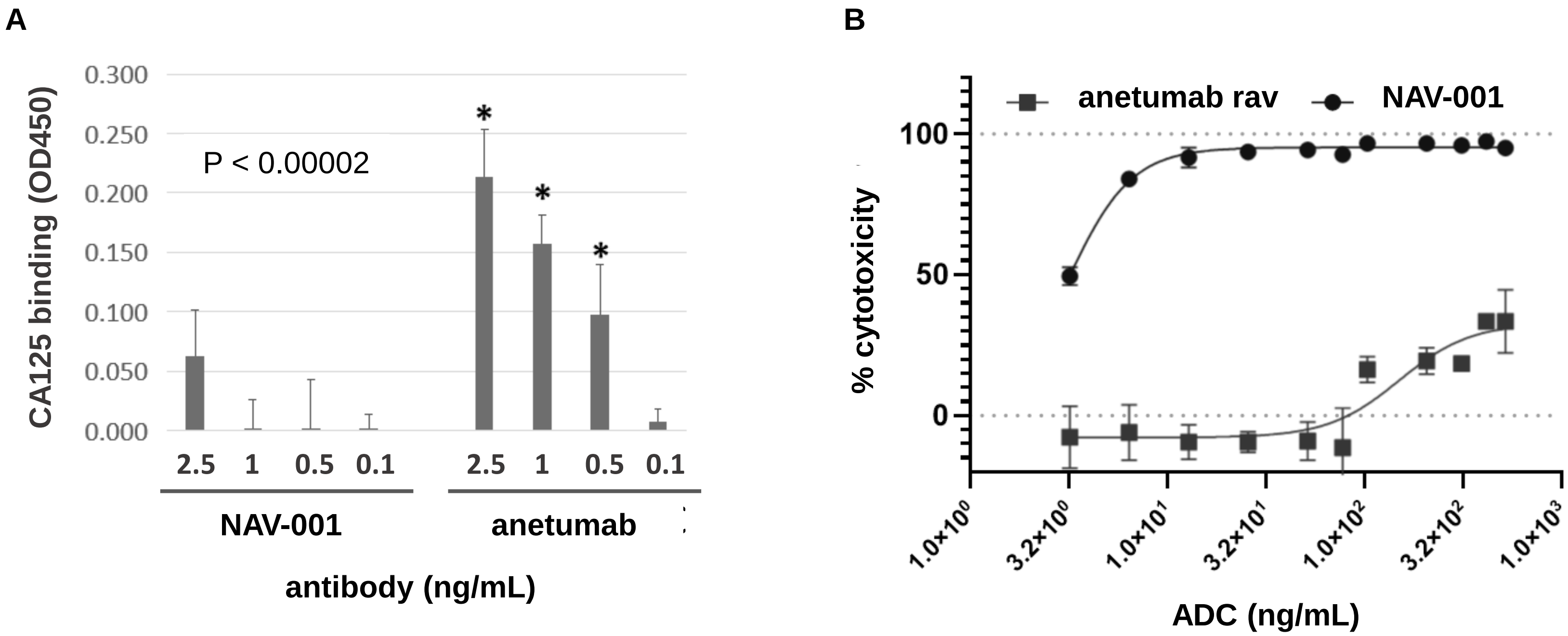

Supplement: S3 Fig — ELISA antibody-CA125 binding assays showed that the MF-T antibody (anetumab) was significantly bound by MUC16/CA125 in contrast to NAV-001 (panel A) (P < 0.00002) and was less effective in killing MSLN-expressing NCI- N87 target cells than NAV-001-PNU when in ADC format (anetumab ravtansine) (panel B). All data represent a minimum of triplicate experiments. (PDF) [file pone.0285161.s003.pdf]
